# Fiber-Reinforced Equibiaxial Dielectric Elastomer Actuator for Out-of-Plane Displacement

**DOI:** 10.3390/ma17153672

**Published:** 2024-07-25

**Authors:** Simon Holzer, Stefania Konstantinidi, Markus Koenigsdorff, Thomas Martinez, Yoan Civet, Gerald Gerlach, Yves Perriard

**Affiliations:** 1 Integrated Actuators Laboratory, Ecole Polytechnique Fédérale de Lausanne, Rue de la Maladière 71b, 2000 Neuchâtel, Switzerlandyoan.civet@epfl.ch (Y.C.); yves.perriard@epfl.ch (Y.P.); 2 Institute of Solid-State Electronics, Faculty of Electrical and Computer Engineering, Dresden University of Technology, Mommsenstraße 15, 01069 Dresden, Germany; markus.koenigsdorff@tu-dresden.de (M.K.); gerald.gerlach@tu-dresden.de (G.G.)

**Keywords:** fiber reinforcement, soft actuators, dielectric elastomer actuators, haptic interfaces

## Abstract

Dielectric elastomer actuators (DEAs) have gained significant attention due to their potential in soft robotics and adaptive structures. However, their performance is often limited by their in-plane strain distribution and limited mechanical stability. We introduce a novel design utilizing fiber reinforcement to address these challenges. The fiber reinforcement provides enhanced mechanical integrity and improved strain distribution, enabling efficient energy conversion and out-of-plane displacement. We discuss an analytical model and the fabrication process, including material selection, to realize fiber-reinforced DEAs. Numerical simulations and experimental results demonstrate the performance of the fiber-reinforced equibiaxial DEAs and characterize their displacement and force capabilities. Actuators with four and eight fibers are fabricated with 100 μm and 200 μm dielectric thicknesses. A maximal out-of-plane displacement of 500 μm is reached, with a force of 0.18 N, showing promise for the development of haptic devices.

## 1. Introduction

Artificial muscles based on various technologies such as pneumatic, thermal or electric systems have emerged in soft robotics and related research fields [1,2,3]. Among these different technologies and systems, dielectric elastomer actuators (DEAs) are interesting due to their ability to provide high strain and strain rates, making them promising candidates in various research fields like biomedicine [4,5,6] and haptics [7,8]. However, their performance is often limited by strain distributions and reduced mechanical stability [9].

Circular DEAs have garnered attention for their high-performance actuation. Often, a circular electrode is used to generate an in-plane deformation [10]. An interesting alternative displacement is in the out-of-plane direction to enhance functionality of DEA technology and to advance its broad applicability in soft robotics [11,12,13]. To do so, DEAs are usually integrated into more complex systems so that the targeted out-of-plane displacement is generated. These systems consist, for example, of springs and are, for example, conical [14,15]. Conically shaped circular DEAs were explored in recent studies, showcasing their ability to generate large strokes and high work output along the out-of-plane axis [14,16,17]. In general, different designs for out-of-plane displacement capabilities enable a broader range of motion and a higher-force output, although often in combination with more complex systems.

Fiber-reinforced DEAs (FRDEAs), inspired by biological tissues and muscles, are a promising approach to enhance the performance of actuators [18,19]. By embedding fibers within the DEA, the soft properties of the dielectric elastomer are maintained, while the fibers enforce different strain distributions, leading to more efficient actuators. For example, fiber reinforcement introduces direction-dependent characteristics into DEAs that can guide and constrain movement effectively among different directions [20]. Especially for uniaxial DEAs, modeling and experimental studies have been carried out in previous works [21].

In this paper, the use of fibers is considered to induce out-of-plane displacement using equibiaxial pre-stretched DEAs. The anisotropic properties of fibers are modeled, providing insights into the behavior of fiber-reinforced DEAs under different loading conditions. A numerical simulation is used to achieve further insight into the material behavior and the possible strain and displacement patterns compared with the displacement measurements. In addition, the displacement and force outputs of the DEAs are measured to show the feasibility of the approach for applications in the field of haptics. This paper is structured as follows: Section 2 provides an overview of the theoretical framework for modeling fiber-reinforced DEAs, including discussions on material properties and modeling approaches. In Section 3, we explain the fabrication process in detail, encompassing material selection, manufacturing techniques and embedding fibers within the actuator structure. In addition, the measurement setups are introduced. Section 4 presents the results of our numerical simulations and experimental validations, offering insights into the performance of fiber-reinforced DEAs. Finally, Section 5 discusses the implications of our findings, explores a potential application in soft robotics and outlines avenues for future research and development.

## 2. Modeling of Anisotropic Circular DEAs

**Figure 1:**
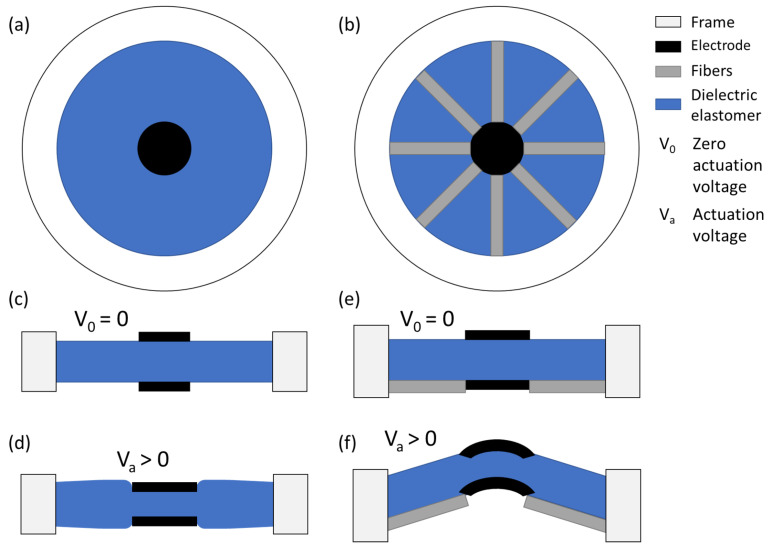
Circular actuator design without (**a**) and with fibers (**b**) and its corresponding function without (**c**,**e**) and with applied voltage ($V_{A}$) (**d**,**f**).

The goal is the development of an analytical model for a circular fiber-reinforced DEAs with radially arranged fibers. To do so, a circular DEA without fibers (Figure 1a) is modeled in Section 2.1, followed by the addition of fibers to the DEA (Figure 1b) in Section 2.3. The functionality of the DEA without fibers is shown in Figure 1c,d, and the functionality and deformation of the DEA with fibers are presented in Figure 1e,f, respectively.

### 2.1. Circular DEAs

Due to the actuation of the DEA with a high voltage, an electric field is induced between the two electrodes, which leads to compressive Maxwell stress along the thickness of the DEA. The generated deformation due to the Maxwell stress for a circular DEA without fibers is similar in plane, as illustrated in Figure 1c,d. The Maxwell stress along the thickness of the DEA can be calculated by the following equation:

(1) $$p=-\frac{\varepsilon_{0}\varepsilon_{r}V^{2}}{l_{z}^{2}}=-\varepsilon_{r}E^{2},$$

where *V* is the actuation voltage, $l_{z}$ is the dielectric elastomer thickness, $\varepsilon_{0}$ is the vacuum permittivity, $\varepsilon_{r}$ is the relative permittivity and *E* is the electric field (such as $E=V/l_{z}$), as the voltage is only applied in the z direction.. This concept was first used for DEAs by Pelrine et al. [22,23]. The main idea for the model presented in this paper is inspired by [24,25]. For further considerations, the cylinder coordinates (r,θ,z) are used to describe the behavior of a DEA. We assume that the dielectric elastomer fulfills the following behavior:

1. The material is incompressible;
2. The deformation in the rθ-plane is homogeneous.

Due to the incompressibility of the material, the volume stays constant, and we can define the $\lambda_{z}$ stretch with the help of stretches $\lambda_{r}$ and $\lambda_{\theta }$ in directions *r* and θ as follows:

(2) $$\lambda_{r}\cdot \lambda_{\theta }\cdot \lambda_{z}=1\Rightarrow \lambda_{z}=\frac{1}{\lambda_{r}\lambda_{\theta }}.$$

Due to the homogeneous material along the rθ plane, we can also simplify the stretches in the center of the device (active area) in the *r* and θ directions as follows:

(3) $$\lambda_{r}=\lambda_{\theta }=\lambda .$$

Using the chosen coordinates, the assumptions and the stretches in the main directions ($\lambda_{r}$ and $\lambda_{\theta }$), the nominal electric field ($\tilde{E}$) can be determined as follows:

(4) $$E=\lambda_{r}\lambda_{\theta }\tilde{E}=\lambda^{2}\tilde{E}$$

With reference to Suo [24], the equation of state for a DEA is defined as follows:

(5) $$s_{i}=\frac{\partial W(C,\tilde{E})}{\partial \lambda_{i}}.$$

where *W* is the total strain energy density containing the mechanical strain component ($W_{s}$) and the electrical component ($W_{E}$). Using this separation, the strain energy density can be written as follows:

(6) $$W\left(C,\tilde{E}\right)=W_{s}\left(C\right)+W_{E}\left(C,\tilde{E}\right),$$

where **C** is the Cauchy–Green deformation tensor and $\tilde{E}$ is the nominal electric field defined by Equation (4). For isotropic materials like dielectric elastomers, the strain energy function is defined as a function of the invariants of the tensor comprising $I_{1}$, $I_{2}$ and $I_{3}$. They are defined as follows:

(7) $$I_{1}=tr\left(C\right)=\lambda_{r}^{2}+\lambda_{\theta }^{2}+\lambda_{z}^{2},$$

(8) $$I_{2}=\frac{1}{2}\cdot \left[tr{\left(C\right)}^{2}-tr\left(C^{2}\right)\right]=\lambda_{r}^{2}\lambda_{\theta }^{2}+\lambda_{\theta }^{2}\lambda_{z}^{2}+\lambda_{r}^{2}\lambda_{z}^{2},$$

(9) $$I_{3}=det\left(C\right)=\lambda_{r}^{2}\lambda_{\theta }^{2}\lambda_{z}^{2}.$$

Because of the convenient modeling capability of the Yeoh model for elastomers and rubbers, especially those containing small particles such as carbon black [26], the Yeoh model is used to describe the strain energy density function ($W_{s}$) [27] as follows:

(10) $$W_{s}\left(C\right)=W_{s}\left(I_{1}\right)=\sum_{i=3}^{3}C_{i0}{\left(I_{1}-3\right)}^{i},$$

where the $C_{i0}$ parameters can be determined experimentally.

To determine the mechanical strain energy density ($W_{s}$), the first invariant of the Cauchy–Green deformation tensor simplified by the given assumptions is expressed as follows:

(11) $$I_{1}=\lambda_{r}^{2}+\lambda_{\theta }^{2}+\frac{1}{\lambda_{r}^{2}\lambda_{\theta }^{2}}.$$

This leads to the following adapted strain energy density function:

(12) $$W_{s}\left(C\right)=W_{s}\left(\lambda \right)=\sum_{i=3}^{3}C_{i0}{\left(2\lambda^{2}+\frac{1}{\lambda^{4}}-3\right)}^{i}.$$

By inserting this strain energy density function in Equation (5), we obtain the nominal stress in *r* direction as follows:

(13) $$s_{r}=\frac{\partial W_{s}\left(C\right)}{\partial \lambda_{r}}+\frac{\partial W_{E}\left(C,\tilde{E}\right)}{\partial \lambda_{r}},$$

with

(14) $$W_{E}\left(\lambda_{r},\lambda_{\theta },\tilde{E}\right)=-\frac{\varepsilon_{0}\varepsilon_{r}}{2}{\tilde{E}}^{2}{\left(\lambda_{r}\lambda_{\theta }\right)}^{2}.$$

### 2.2. Pre-Stretch and Influence of Passive Regions

The pre-stretch ($\lambda_{pre}$) of the dielectric elastomer leads to an additional force ($F_{P}$); hence, the radial stress without electrical actuation is expressed as follows:

(15) $$s_{r}=\frac{F_{P}}{2\pi tR}=\frac{\partial W_{s}\left(C\right)}{\partial \lambda_{r}}+\frac{\partial W_{E}\left(C,\tilde{E}\right)}{\partial \lambda_{r}},$$

where *R* is the initial radius of the electrode and *t* is the initial thickness of dielectric membrane.

The influence of the surface coverage of the actuator can be evaluated as in [25]. An equibiaxial stress state is assumed, along with the same stiffness over the whole dielectric layer for both the active and passive regions. The stretch ratios over active ($\lambda_{a}$) and passive ($\lambda_{p}$) areas can then be defined separately as follows:

(16) $$0=\frac{\partial W(\lambda_{r,\theta }=\lambda_{A})}{\lambda_{r}}+\frac{\partial W_{E}\left(C,\tilde{E}\right)}{\partial \lambda_{r}}-s_{r}\left(\lambda_{P}\right),$$

where $\lambda_{A}=\lambda_{a}\lambda_{pre}$ and $\lambda_{P}=\lambda_{p}\lambda_{pre}$ correspond to the stretches in the active and passive regions, respectively. Given that the stretch ratio in the passive area ($\lambda_{P}$) decreases when the stretch in the active area ($\lambda_{A}$) increases during actuation, the following relation can be written, where (*R*) denotes the coverage ratio of the actuator:

(17) $$R\lambda_{a}+\left(1-R\right)\lambda_{p}=1.$$

Through the stretch in the active area, it is then possible to determine the stretch in the passive area.

### 2.3. Out-of-Plane Displacement Prediction

The fiber-reinforced actuator geometry shown in Figure 1e,f is considered for determination of the out-of-plane displacement. The following assumptions are used:

1. No strain on polydimethylsiloxane (PDMS) covered by fibers;
2. The fibers are rigid and non-stretchable;
3. No force hindering the electrodes and active area from out-of-plane movement.

Solving Equations (15)–(17) allows for determination of the strain in the radial direction ($\epsilon_{r}$). The corresponding displacement in the radial direction can then be written as follows:

(18) $$u_{r}=\epsilon_{r}r_{a},$$

with $r_{a}$ being the active electrode radius. Considering an angle (α) induced by the actuation as in Figure 1, the out-of-plane displacement can then be described as follows:

(19) $$u_{z}=sin\left(\alpha \right)L_{f},$$

where $L_{f}=L_{r}-r_{a}$ is the fiber length and α is the angle formed between the fibers and the plane.

(20) $$L_{r}=cos\left(\alpha \right)L_{f}+r_{a}\lambda_{r}\epsilon_{r}.$$

**Figure 2:**
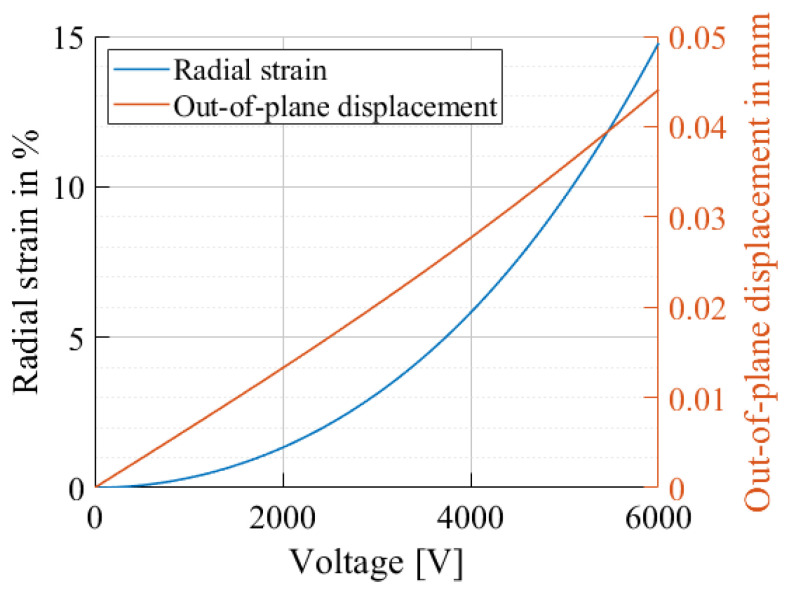
Model results for the radial strain and corresponding out-of-plane displacement as a function of the applied voltage with a coverage ratio of 20%, electrode radius of 5 mm and 1.5 applied equibiaxial pre-stretching.

Equations (19) and (20) can finally be solved to calculate the final out-of-plane displacement. Figure 2 shows the results of the exemplary model for a circular DEA with a coverage ratio of 20%.

### 2.4. Numerical Simulations

The proposed analytical model does not take into consideration the fiber width and the surface covered by fibers. To study their influence, in addition to the analytical model, a numerical simulation is executed using COMSOL Multiphysics 6.0. The selected space dimension is *3D*, the selected physics parameter is electromechanics and the selected study is stationary. The design of the device is sketched in COMSOL Multiphysics 6.0 with the geometry tools, with the designs explained in Section 3.1. For the DEA device, the PDMS and polyethylene terephthalate (PET) materials from the COMSOL library are used. The parameters used for the mechanical model from PDMS are adjusted by Yeoh parameters found in the literature for the equibiaxial stretch of Elastosil 2030 [25]. In the solid mechanics section, the hyperelastic material domain condition is used for the dielectric elastomer. In the electrostatics section, the ground and electric potential boundaries are used to define the electrodes. The mesh was calibrated for general physics, and a predefined size of coarser is used. The study is applied for different actuation voltages. With the simulation, the displacement in z direction of the center is analyzed. A summary of the important settings used for the simulation are specified in Table 1.

## 3. Materials and Methods

### 3.1. Design and Fabrication

**Figure 3:**
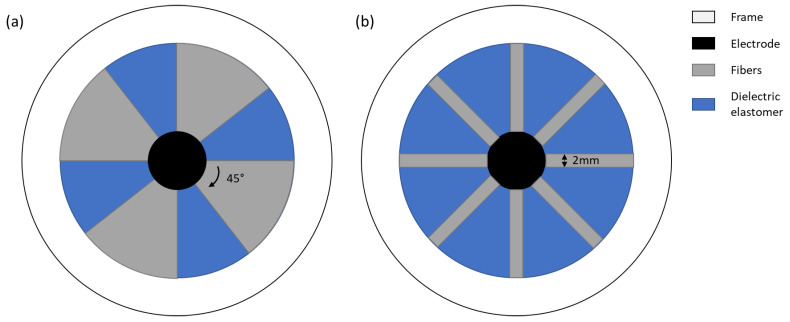
Design of DEA with four (**a**) and eight (**b**) fibers.

The DEA is composed of a dielectric elastomer (Elastosil 2030, 100 μm, Wacker Chemie AG, München, Germany) on which round electrodes with a unstretched diameter of 5 mm are stencil-printed. The DEA is pre-stretched with a factor of 1.5, and the pre-stretch is fixed with the help of a circular frame. The inner diameter of the frame is 30 mm. On one side of the DEA, four or eight fibers are attached (Figure 3).

**Figure 4:**
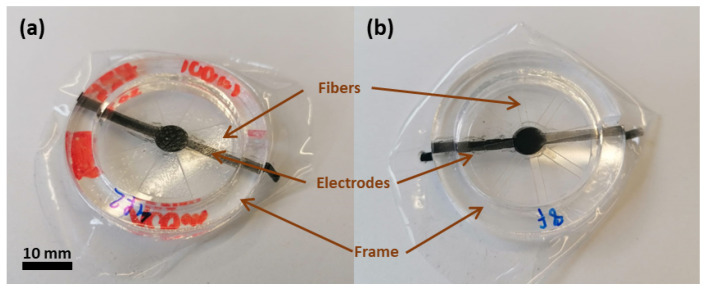
Fabricated DEAs with four (**a**) and eight (**b**) fibers.

The electrodes are based on a carbon composite containing carbon black (Ketjenblack EC-600JD Conductive Carbon Black, Nouryon, Amsterdam, The Netherlands), a silicone matrix (Silbione LSR 4305, Elkem, Oslo, Norway) and solvent (2-Propanol, Sigma-Aldrich Merk, Burlington, VT, USA). The detailed recipe with electrode characterization is given in [28]. For the fabrication, a mask is laser-cut into a circular shape. The electrode mixture is stencil-printed onto the dielectric elastomer with a film applicator (ZAA 300, Zehnter Testing Instruments, Sissach, Switzerland) and thermally cured at 80 °C for 4 h. In order to apply the electrode on the other side of the dielectric elastomer, a thin layer of silicone glue (LSR 4305) is applied on the first electrode while maintaining the electrical connections. The elastosil film is transferred onto a new PET substrate with the electrode facing down. Lastly, the second electrode is screen-printed and thermally cured. Next, pre-stretching is applied to the fabricated DEA. The fibers were fabricated in the laboratory from PET sheets to enable better control of manufacturing parameters. Laser engraving was used to adjust the length and shape of the fibers, which were then bonded to the DEA. A frame is kept around them in order to maintain their alignment and act as support during the fabrication process. Instant adhesive glue for rubbers is then applied on the fibers (ARclear 8932EE, Adhesives Research, Glen Rock, NJ, USA), and the fibers are placed on the top of the pre-stretched actuators. Two different fiber designs are fabricated—one with four and one with eight fibers. The resulting actuators are shown in Figure 4.

### 3.2. Setup and Characterization

**Figure 5:**
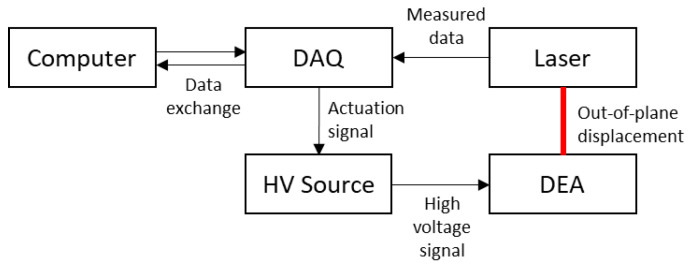
Measurement setup to determine the out-of-place displacement.

In order to study the influence of the fibers, the DEAs are actuated with voltages varying from 0 to 7 kV (TREK 10/10B-HS, Advanced Energy, Denver, CO, USA). Cyclic measurements were performed with voltages varying from 0 to 8 kVs with steps of 1 kV. For every voltage, the actuator underwent 15 cycles at a frequency of 0.5 Hz and a duty cycle of 50 %. The high-voltage source is controlled by a data acquisition device (DAQ DEVICE MULTIFUNC I/O USB 2.0, National Instruments, Austin, TX, USA). The displacement of the DEA is measured by monitoring the profile of the electrodes with the help of a laser (Gocator, Keyence, Osaka, Japan). The actuation of the DEA is controlled and recorded by a MATLAB R2022b script. The setup is illustrated in Figure 5.

**Figure 6:**
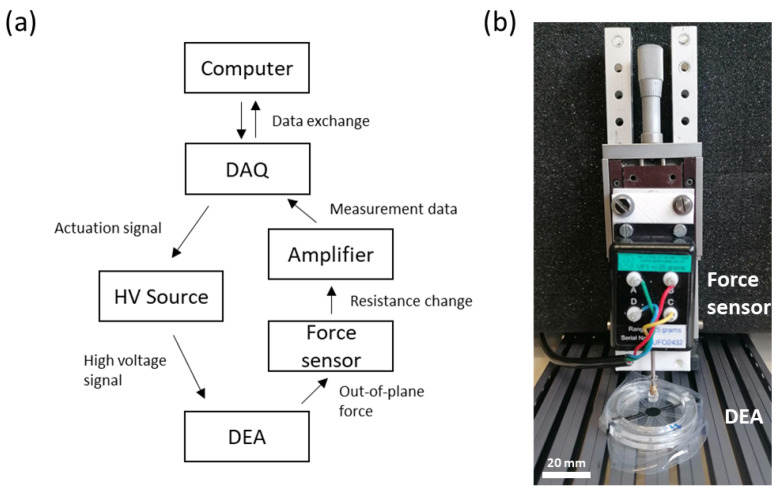
(**a**) Measurement setup to determine the out-of-place force. (**b**) Force sensor with DEA.

In Figure 6, the setup used for force sensing is shown. It consists of a Trek (TREK 10/10B-HS, Advanced Energy, Denver, CO, USA), which is used for the actuation of the DEA. In addition, a force cell (UF1 +/− 25 g, Applied Measurements Ltd, Aldermaston, UK) is used to determine the out-of-plane force. The cell is mounted on a movable stage to adjust the tip on the surface of the DEA. The force cell is connected to the intelligent panel mount (Panel Mount Display IPM 650, Futek, Irvine, CA, USA), which is further connected to a data acquisition device (DAQ, NI DAQPad-6259 Pinout, National Instruments, Austin, TX, USA).

## 4. Results

### 4.1. Simulation Results

The simulation was carried out for the two different designs, namely a design with four fibers and a design with eight fibers (Figure 3). The numerical simulation results in a bigger displacement for the design with four fibers. The maximum out-of-plane displacement is around 1.5 mm. For the design with eight fibers, the maximum displacement is circa 1.25 mm. The displacement is nearly zero for low voltages (<1 kV). At around 1 kV, there is a jump in displacement. After the abrupt increase, the displacement rises approximately linearly for higher voltages.

**Figure 7:**
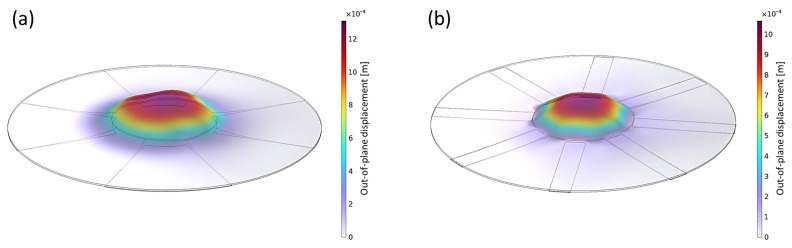
Displacement of fiber-reinforced DEA at 6 kV actuation with (**a**) four and (**b**) eight fibers.

The displacements at 6 kV are shown graphically in Figure 7. The displacement in the middle of the device for a DEA design with four fibers is less homogeneous than that of the eight-fiber DEA. To decrease uncertainty due to imprecise behavior in the middle of the simulation (blowing-up effect), which was not observed in experiments, the displacement at the tip of the fiber is determined and compared in Section 5.

### 4.2. Experiments

#### 4.2.1. Out-of-Plane Displacement

**Figure 8:**
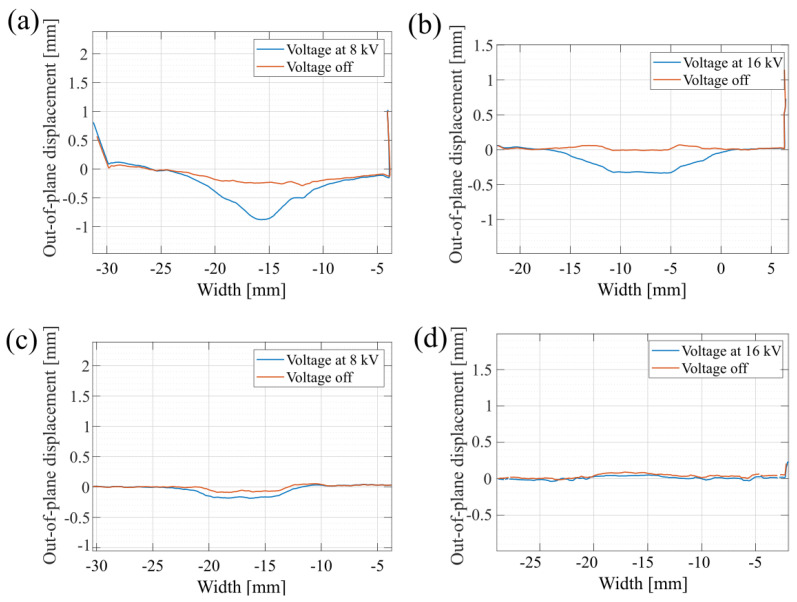
Out-of-plane displacement measured across the diagonal of DEAs with (**a**) four fibers, 100 μm dielectric thickness, operated at 8 kVs; (**b**) eight fibers, 200 μm dielectric thickness, operated at 16 kVs; (**c**) eight fibers, 100 μm dielectric thickness, operated at 8 kVs and (**d**); without fibers, 200 μm dielectric thickness, operated at 16 kVs.

For all actuators, the out-of-plane displacement is characterized using a 2D laser sensor (Gocator, Keyence, Osaka, Japan). Such measurements allow the actuation profile to be obtained over the whole actuator. Figure 8 illustrates the actuation profile for actuators with four and eight fibers and with dielectric thicknesses of 100 and 200 μm. A reference actuator without any fibers and with a 200 μm dielectric thickness is also characterized. The actuators are operated with voltages up to 8 kVs for the 100 μm thickness and with voltages up to 16 kVs for the 200 μm thickness. The center of the actuator induces an out-of-plane displacement in a conical and flat profile, depending on the length of the fibers and their overlap on the electrode surface. It can be noted that the peaks located at the extremities of the actuator membrane correspond to the polymethyl methacrylate (PMMA) frame acting as a support for the actuator.

**Figure 9:**
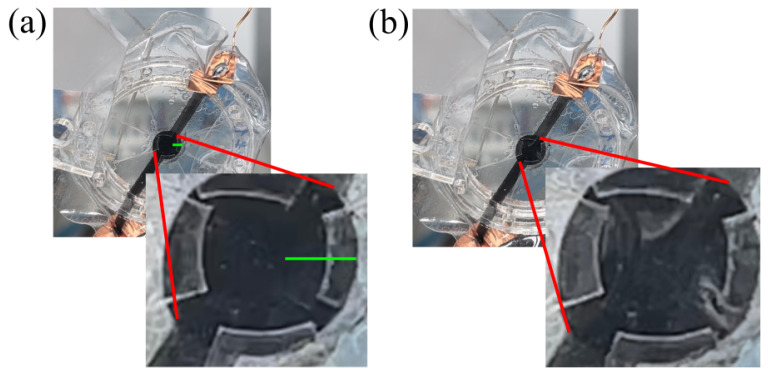
Illustration of DEA actuation for the actuator with four fibers and a 100 μm dielectric membrane. (**a**) Membrane at rest; (**b**) applied voltage of 8 kVs. Scale bar (green): 3 mm.

A maximal out-of-plane displacement of 500 μm is reached for the actuator with four fibers and a 100 μm dielectric membrane. Video captures of the actuation are shown in Figure 9.

**Figure 10:**
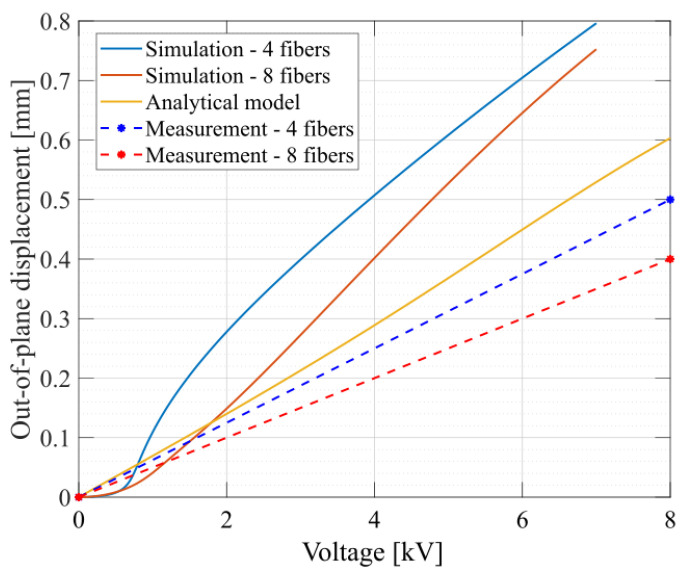
Comparison of simulation, model and measurement results for the maximal out-plane-displacement reached with FRDEAs composed of four or eight fibers as a function of the actuation voltage.

In Figure 10, the results for the displacement at the top edge of the fibers obtained by the numerical simulation, the analytical model and the measurements are presented. The behavior was simulated until 7 kV, after which instabilities occurred. The analytical calculation, as well as the measurements, are determined for actuation voltages up to 8 kV. As the analytical model does not take into consideration the fiber geometry, a single plot is produced. However, it can be noticed that the actuation amplitude varies depending on the number of fibers, similarly to the simulation predictions.

**Figure 11:**
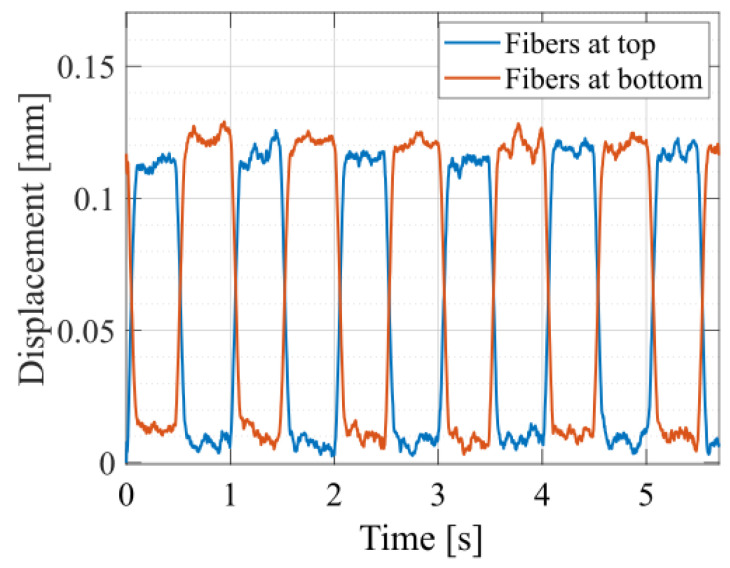
Time-dependent out-of-plane displacement of fiber-reinforced circular DEAs with eight fibers, a 100 μm dielectric thickness and voltage pulses of 7 kVs. The fibers are placed on the top or on the bottom of the actuator.

In order to study the influence of gravity, the actuators are also tested in dynamic actuation cycles, with the fibers being placed on top of the electrode and with the fibers placed on the bottom of the electrode after reversing the actuator. For this, the displacement in the center of the membrane is measured by placing a 1D laser sensor in the center of the electrode. As seen in Figure 11, the displacement magnitude for these two configurations is similar. As expected, there is a sign difference, as the out-of-plane displacement always occurs on the side of the fibers, meaning that in one case, the membrane moves downwards (red curve, for top fiber configuration), and in the second case, the membrane moves upwards (blue curve, for fibers placed at the bottom).

#### 4.2.2. Out-of-Plane Force

**Figure 12:**
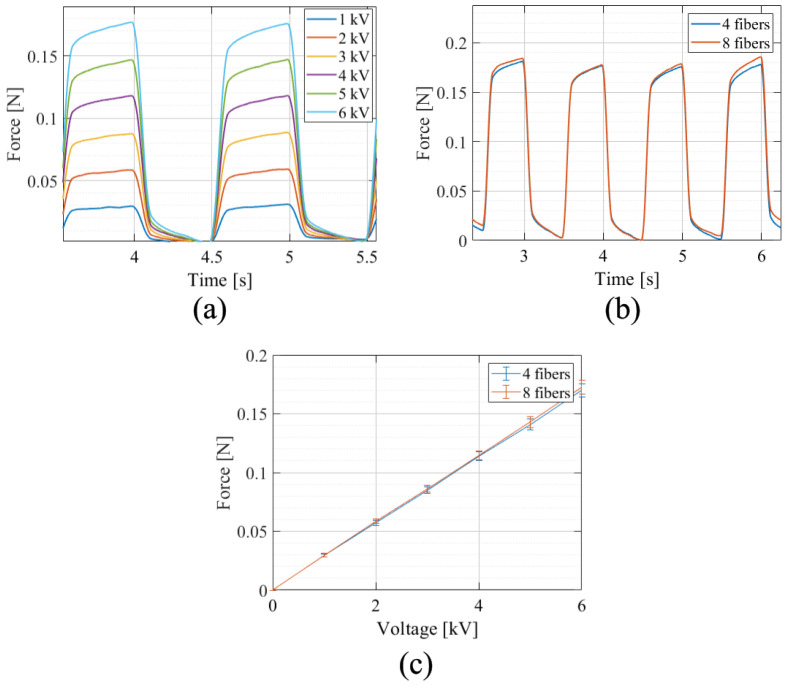
Time-dependent out-of-plane force of fiber-reinforced actuators as a function (**a**) of applied voltage for a DEA with four fibers and (**b**) of a number of fibers. (**c**) Out-of-plane force versus applied voltage for actuators with four and eight fibers.

The recorded forces for different voltages is plotted are Figure 12. As can be seen, the force increases linearly with the applied voltage. The measured force for four and eight fibers is similar and reaches 0.18 N, with 0.13% difference in the force between the two configurations at 6 kVs.

## 5. Discussion

### 5.1. Analysis of Proposed Device

The out-of-plane displacement of fiber-reinforced equibiaxial actuators can be predicted by an simple analytical model, taking into consideration only the electrode surface coverage and the pre-stretch. Although out-of plane strains occur, the proposed analytical model did not yet take into consideration the fiber width and the surface covered by fibers and, thus, does not allow us to optimize these parameters. With the help of numerical simulation, various designs were tested with respect to their out-of-plane displacement. In particular, two different designs (four and eight fibers) were simulated. A non-linear displacement occurs up to a voltage of circa 1 kV according to the simulation. At higher voltages, a sudden increase in displacement takes place, followed by an almost linear increase at voltages of more than 2 kV. This simulated behavior promises good control of actuation, especially in the linear region between 1.5 kV and 7 kV.

The simulations predicted a higher displacement for DEAs compared to the displacement actually measured during the experiments. The values from the measurements are around 50–65% of the simulated values. We assume that this difference is caused by several reasons. A first possibility is that the fibers loosen slightly from the glue due to the stretching at the edge of the electrodes, which leads to a reduced fixed constraint in the in-plane direction and, thus, to a reduced displacement in the out-of-plane direction. A second possibility is the integration of pre-stretching into the numerical simulation. This effect is not taken into account in COMSOL Multiphysics but can have an influence on the behavior.

A third reason is the hyperelastic material parameters used for the simulation. These were determined for biaxial behavior of an Elastosil 2030 sheet. However, the values change slightly due to the addition of electrodes and insulating layers; therefore, what was not included in the material parameters and the simulation is only an approximation. A fourth influence can be caused by slight deviation of the ideal laser position. If possible, the laser was positioned in the center of the electrode in order to determine the maximum displacement, but a certain degree of inaccuracy can occur during positioning. To evaluate the influence of the fiber reinforcement of the moved DEAs, their out-of-plane displacement was compared to actuators without any fibers. A maximal out-of-plane displacement of 500 μm was found for the actuator with four fibers and 100 μm for a dielectric membrane. Pointed conical profiles were observed for actuators when the fibers overlapped with the electrode surface, whereas a flat profile was observed for actuators where the fibers only covered the passive area. An influence of gravity on the behavior could not be determined. The out-of-plane behavior of the DEAs remains the same, regardless of how the actuator is rotated.

In addition to the out-of-plane displacement, the generated out-of-plane force was also measured for voltages in the range of 1–6 kV. Values in the order of magnitude of up to 0.18 N could be determined. The generated force is approximately the same for both fiber designs and increases linearly with voltage. Compared to the displacement measurements, the data for the generated force are less noisy and show better linear behavior between actuation voltage, displacement and force. The observed difference between the two actuators with different fiber counts in terms of their generated force is negligible.

### 5.2. Possible Applications of FRDEAs

One possible application for fiber-reinforced equibiaxial DEAs lies in the field of haptic devices. Haptic devices are often associated with consumer electronics. Examples include, on the one hand, touchscreens, in which the haptic feedback is integrated to obtain improved feedback [29], and, on the other hand, devices in connection with virtual reality that offer the possibility to improve the imitation of real-life situations like the sense of touch [30]. This novel approach is interesting and can help to develop new training approaches for different professional groups. One possibility is in the training of future surgeons and doctors, where training with VR offers the opportunity to train in a way that is safe for patients. It also provides a training opportunity for rare procedures that are otherwise rarely practiced.

**Figure 13:**
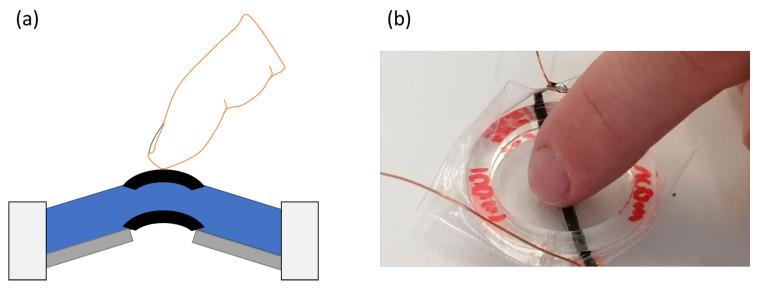
(**a**,**b**) Illustration of an application in haptics. The force resulting from DEA actuation can be felt by a finger placed on the electrode.

In order to determine the feasibility of such a haptic device, threshold values for haptic sensing are analyzed. In general, a well-defined threshold is not possible, as the sense of touch is very individual. An approximate threshold value for the minimum detectable displacement force is 30 μm for the tip of the thumb [31], and that for the force of the finger tip is 10 mN at a frequency of 5–10 Hz [32]. Electroactive polymer devices for haptic applications have shown that a displacement of 450 μm and a force of 14 mN are attainable and sufficient for haptic sensing [33]. These conditions are all fulfilled by the fiber-reinforced equibiaxial DEA proposed in this work. Tests to determine at which values the limits of the haptic perception for different body parts like the finger tip (Figure 13) or other areas of the body will be carried out in the future.

## 6. Conclusions

In this study, we present a novel approach for fiber-reinforced DEAs, emphasizing material selection and manufacturing techniques to optimize deflection and force [21]. Through a combination of analytical and numerical simulations and experimental validations, we investigated the efficacy of these fiber-reinforced equibiaxial DEAs and characterized their displacement capabilities. Notably, our focus lies in developing comprehensive models that capture the out-of-plane behavior of these actuators resulting from the anisotropic behavior of the fiber-reinforced membrane. In future work, this anisotropy can also be considered in the constitutive models to account for the direction-dependent properties of the actuator. This modeling approach draws inspiration from the anisotropic nature of biological tissues and muscles, aiming to accurately represent the complex mechanical behavior of fiber-reinforced DEAs [34].

## Figures and Tables

**Table 1:** Numerical simulation parameters.

Category	Subcategory	Parameter	Value	Unit
Materials	PDMS	Relative permittivity	2.8	-
		Density	970	kg/m^3^
		Yeoh coefficient C1 [25] Yeoh coefficient C2 [25] Yeoh coefficient C3 [25]	178.4 −6.0 4.2	kPa kPa kPa
	PET	Relative permittivity	2.9	-
		Density	1375	kg/m^3^
		Young’s modulus	2950	MPa
		Poisson’s ratio	0.34	-
Physics	Solid mechanics	Hyperelastic material	Yeoh model	
		Prescribed displacement	1.5	
	Electrostatics	Ground	0	kV
		Electric potential	0–6	kV
Mesh	Size	Element size	coarser	
	Generator	Free tetrahedral		
Study	Step 1: Stationary	Add voltage swipe	0–6	kV

## Data Availability

The data presented in this study are available on request from the corresponding author. The data are not publicly available due to privacy or ethical restrictions.

## Abbreviations

The following abbreviations are used in this manuscript:

DEA	Dielectric Elastomer Actuator
FR	Fiber-reinforced
